# Separation of Teeth

**Published:** 1896-03

**Authors:** T. F. Chupein


### Separation of Teeth.

BY T. F. CHUPEIN.

Rubber for separating the teeth is little used on account of its great activity and of its disposition to work its way toward the neck of the tooth, thereby pressing on the gum and causing considerable pain. Yet rubber is made in special forms and used for this purpose still. This style of rubber often gets quite stiff, hard and rotten, making it, when wanted, unfit for use. Rubber-dam is always at hand, is always fresh and always ready for use. If a piece of this be *twisted in a roll between the thumb and fingers* it can be made in any size necessary for the case in hand, and being thus made cylindrical, is in the best form for application. Three, four, five or a dozen turns can be made of a small discarded piece of an inch square to place between the teeth to effect their separation.

Children's Food.—Dr. Allen does not approve of giving little children coarse, hard food to masticate. The temporary teeth are not suited to that purpose from their brief duration, during the early part of which the roots are undeveloped, and the teeth not firmly fixed in the alveolus, while during the latter portion of their retention the roots are being absorbed, the edges having sharp, jagged points not fitted to bear the pressure of excessive mastication. The effort being painful, the child swallows its food as best it can, and the foundation for dyspepsia is early laid.

